# Acute Scrotal Involvement in Kawasaki Disease: A Potential Clinical Clue to Persistent Disease Activity

**DOI:** 10.1155/crpe/4413879

**Published:** 2026-07-30

**Authors:** Yuma Inui, Katsuyuki Yokoi, Chihaya Sumida, Masato Itano, Natsuki Hatakawa, Takanori Suzuki, Hidetoshi Uchida, Kazuyoshi Saito, Yoshiki Kawamura

**Affiliations:** ^1^ Department of Pediatrics, Okazaki Medical Center, Fujita Health University, Okazaki, Aichi, Japan, fujita-hu.ac.jp; ^2^ Department of Pediatrics, Kariya Toyota General Hospital, Kariya, Aichi, Japan, toyota-kai.or.jp; ^3^ Department of Pediatrics, School of Medicine, Fujita Health University, Toyoake, Aichi, Japan, fujita-hu.ac.jp

**Keywords:** acute scrotum, coronary artery aneurysm, Kawasaki disease, vascular inflammation

## Abstract

Kawasaki disease (KD) is an acute vasculitis and the leading cause of acquired heart disease in children. Coronary artery aneurysms (CAAs) arise from vascular inflammation during the acute phase; however, in some patients, inflammation may persist despite treatment, and reliable clinical indicators of ongoing disease activity remain limited. Acute scrotal involvement is a rare manifestation of KD, and its clinical significance has not been well established. Here, we report a case of a previously healthy 49‐month‐old boy who presented with 4 days of fever and the five principal features of KD. He was initially treated with intravenous immunoglobulin, aspirin, and prednisolone, resulting in transient improvement. On Day 7 of illness, he developed recurrent fever and right inguinal pain. Fever persisted on Day 8, accompanied by recurrent lip erythema and worsening inflammation, prompting a second dose of intravenous immunoglobulin. His fever improved on Day 9, when right‐sided scrotal erythema and swelling became evident. Ultrasonography revealed a right hydrocele with preserved blood flow and no epididymal torsion. Echocardiography on Day 11 demonstrated medium‐sized bilateral CAAs, which were subsequently confirmed by coronary computed tomography. The scrotal findings resolved completely within 1 month. This clinical course suggests that acute scrotal involvement may represent a potential clinical clue to persistent disease activity in KD, warranting careful reassessment for evolving coronary artery involvement.

## 1. Introduction

Kawasaki disease (KD) is an acute febrile illness that primarily affects infants and represents the leading cause of acquired heart disease in children in developed countries [1]. A major complication of KD is the development of coronary artery aneurysms (CAAs), which arise from inflammatory infiltration of the arterial wall, leading to structural disruption and subsequent dilation [2]. Early and appropriate treatment aimed at suppressing vascular inflammation is therefore essential to prevent CAA formation [1]. However, in some patients, vascular inflammation may persist despite therapy, and reliable clinical indicators of ongoing disease activity remain limited. An acute scrotum, defined as the sudden onset of scrotal pain, swelling, and erythema [3], is a rare but reported manifestation of KD [4–13]. This presentation may reflect underlying vascular inflammation or increased vascular permeability; however, its clinical significance has not been well characterized. Here, we report a case of KD, in which acute scrotal involvement developed following an initial transient clinical improvement and was subsequently associated with progression of CAAs. Moreover, we review previously reported cases to explore the significance of acute scrotal manifestations as a potential clinical clue to persistent disease activity in KD.

## 2. Case Presentation

**FIGURE 1:**
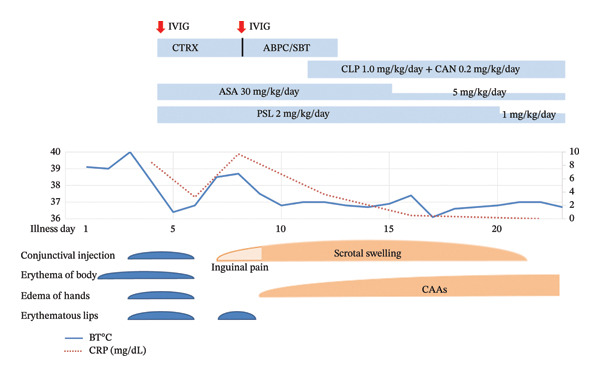
Clinical course of the patient. IVIG, intravenous immune globulin; CTRX, ceftriaxone; ABPC/SBT, ampicillin/sulbactam; CLP, clopidogrel; CAN, candesartan; CAAs, coronary artery aneurysms.

A previously healthy 49‐month‐old boy was admitted with a 4‐day history of fever, conjunctival injection, erythematous lips, truncal erythema, and swelling and erythema of the hands, fulfilling the diagnostic criteria for KD. Figure 1 summarizes the patient’s clinical course and laboratory data during the acute phase. Admission laboratory studies showed the following: white blood cell (WBC) count, 14.9 × 10^3^/μL (86.7% neutrophils); hematocrit, 36.7%; platelet count, 402 × 10^3^/μL; sodium, 126 mmol/L; aspartate aminotransferase levels, 38 U/L; alanine aminotransferase, 20 U/L; total bilirubin, 0.5 mg/dL; albumin, 3.4 g/dL, C‐reactive protein (CRP), 8.39 mg/dL; and D‐dimer 2.9 μg/mL. Dipstick urinalysis revealed negative leukocyte esterase, hematuria (1+), and proteinuria (±). Echocardiography revealed the following coronary artery dimensions and Z‐scores: right coronary artery (RCA), 2.1 mm (*Z* = 1.12), left main trunk (LMT), 2.0 mm (*Z* = −0.12), left anterior descending artery (LAD), 1.4 mm (*Z* = −0.95), and left circumflex artery (LCX), 1.5 mm (*Z* = 0.03).

Given a Kobayashi score 14 of 6, indicating high risk for intravenous immunoglobulin (IVIG) resistance, he received IVIG 2 g/kg over 12 h, aspirin 30 mg/kg/day, and prednisolone 2 mg/kg/day. Ceftriaxone was administered to cover possible bacterial infection. Fever resolved temporarily on illness day 5, with improved inflammatory markers (CRP, 3.21 mg/dL). On illness day 7, recurrent fever and intermittent right inguinal pain developed. On Day 8, fever persisted with recurrent lip erythema; repeat laboratory studies showed CRP 9.7 mg/dL and D‐dimer 4.0 μg/mL.

**FIGURE 2:**
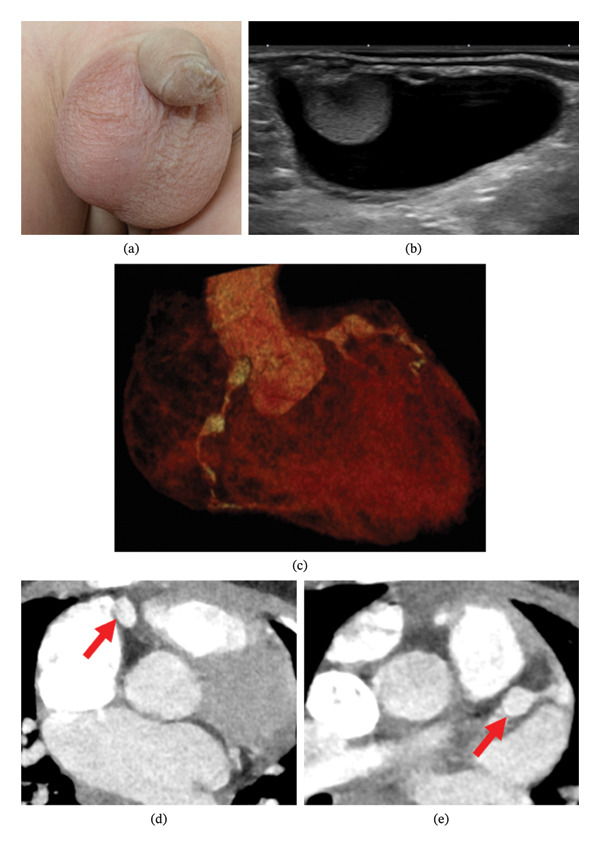
Clinical and imaging findings in the acute phase of Kawasaki disease. (a) Photograph of the scrotum on illness day 9 showing erythema and swelling localized to the right side. (b) Scrotal ultrasonography demonstrating a right‐sided hydrocele. (c) Coronary CT providing an overall view of the coronary arteries and revealing aneurysmal changes in the RCA, LMT, LAD, and LCX. (d) Coronary CT highlighting a coronary artery aneurysm of the RCA (red arrow indicates the aneurysm). (e) Coronary CT highlighting a coronary artery aneurysm of the LAD (red arrow indicates the aneurysm). CT, computed tomography; RCA, right coronary artery; LMT, left main trunk; LAD, left anterior descending artery; LCX, left circumflex artery.

Echocardiography revealed the following coronary artery dimensions and Z‐scores: RCA, 2.0 mm (*Z* = 1.0), LMT, 2.7 mm (*Z* = 2.1), and LAD, 4.0 mm (*Z* = 5.0). In view of these changes, a second dose of IVIG (2 g/kg over 12 h) was administered. Because bacterial infection could not be excluded, the antibiotic regimen was changed from ceftriaxone to ampicillin/sulbactam to broaden antimicrobial coverage, including anaerobic bacteria. His fever improved on illness day 9. On the same day, right‐sided scrotal erythema and swelling became apparent (Figure 2a), explaining the earlier right inguinal pain. Scrotal ultrasonography revealed a right‐sided hydrocele (Figure 2b) without left‐sided abnormalities. The right testicular parenchyma was homogeneous, with preserved peripheral and intratesticular blood flow and no significant asymmetry compared with the left side. No notable bilateral differences were observed between the epididymides. As there were no ultrasonographic findings suggestive of testicular torsion, conservative management was selected. Echocardiography on Day 11 demonstrated progression of the coronary artery lesions, with medium‐sized CAAs involving the RCA (4.9 mm [*Z* = 6.96]), LMT (3.8 mm [*Z* = 4.6]), LAD (4.9 mm [*Z* = 6.9]), and LCX (3.2 mm [*Z* = 3.77]). Clopidogrel and candesartan were initiated. On illness day 15, the aspirin dose was reduced to 5 mg/kg/day after the CRP level had decreased to 0.49 mg/dL. Prednisolone was tapered to 1 mg/kg/day on Day 20. Coronary computed tomography (CT) on Day 22 demonstrated aneurysmal changes in the RCA, LMT, LAD, and LCX (Figure 2c–e). The patient was discharged on Day 23. During follow‐up, the scrotal swelling gradually decreased, with complete remission observed 1 month after the onset of KD. During the clinical course, the peak coronary artery diameters and Z‐scores on echocardiography were as follows: RCA, 6.0 mm (*Z* = 8.6); LMT, 3.8 mm (*Z* = 4.6); LAD, 6.27 mm (*Z* = 7.44); and LCX, 3.2 mm (*Z* = 3.77). At the 8‐month follow‐up, echocardiography showed improvement of the coronary artery lesions, with coronary artery dimensions of RCA, 2.5 mm (*Z* = 2.0); LMT, 3.0 mm (*Z* = 2.5); LAD, 3.5 mm (*Z* = 4.0); and LCX, 1.6 mm (*Z* = 0.2).

## 3. Discussion

An acute scrotum has various causes, including testicular torsion, epididymitis, trauma, incarcerated inguinal hernia, acute idiopathic scrotal edema, and systemic vasculitis, such as Henoch–Schönlein purpura [3]. Acute scrotal involvement has also been reported in patients with KD [4–13]. In the present case, other causes were excluded based on ultrasonographic and clinical findings, and the scrotal symptoms were attributed to KD. KD is classified as a vasculitis primarily affecting medium‐sized arteries, although inflammation can involve small arteries [15]. The testicular artery arises from the abdominal aorta, and a previous report described KD‐associated acute scrotum with findings suggestive of testicular arteritis [16]. In addition, in polyarteritis nodosa, another medium‐vessel vasculitis, painful testicular swelling due to testicular arteritis is a well‐recognized clinical manifestation [17]. These observations suggest that vascular inflammation in KD can extend to the testicular artery, potentially leading to acute scrotal symptoms.

In our review of previously reported cases of KD with acute scrotal involvement, some patients presented with scrotal edema without clear local inflammatory signs (Table 1) [4–13]. This has been attributed to the systemic increase in vascular permeability characteristic of the acute phase of KD. In our case, unilateral scrotal erythema, swelling, and pain suggested that local inflammation, rather than vascular leakage alone, contributed to the symptoms. Marked elevations in WBC and CRP levels supported this hypothesis. Collectively, these findings suggest that acute scrotal involvement in KD may result from a combination of local arteritis‐related inflammation and increased vascular permeability.

**TABLE 1:** Summary of acute scrotal manifestations in Kawasaki disease [4–13].

Report	Patient age (months)	No. criteria fulfilled	Day of KD diagnosis	Treatment	Nonscrotal edema (acute phase)	Day coronary artery abnormalities first observed (site, maximum severity)	Day of acute scrotal manifestation (illness day, laterality)	Diagnosis of scrotal manifestation	Outcome
Connolly and Timmons. [4] 1	72	6	NR	ASA	Right wrist	None	5 (left)	Epididymo‐orchitis	Resolved 9 days later
Kabani et al. [5] 2	10	3	10	IVIG (400 mg/kg/day, duration NR), ASA	NR	10 (LCA, aneurysm of unknown severity)	6 (left)	Hydrocele testis	Resolved 1 week later
3	11	4	8	IVIG (400 mg/kg/× day, duration NR), ASA	Feet	8 (LCA, dilation of 4 mm)	10 (right)	Hydrocele testis	Resolved 1 week later
4	23	4	10	IVIG (400 mg/kg/× day, duration NR), ASA	NR	None	6 (bilateral)	Hydrocele testis	Resolved 2 weeks later
5	36	6	7	IVIG (400 mg/kg/day, duration NR), ASA	NR	None	7 (left)	Hydrocele testis	Resolved later
6	44	4	9	ASA	NR	None	10 (left)	Hydrocele testis	Surgery 2 years later
Pavone et al. [6] 7	18	6	4	2 × IVIG (2 g/kg), ASA	Hands	None	5 (laterality NR)	Scrotal swelling	NR
Leonardi et al. 2013 [7] 8	3	5	no description	2 × IVIG (2 g/kg), ASA, PSL, clopidogrel	Generalized edema	6 (RCA, LCA, LAD; large aneurysms)	After second IVIG (laterality NR)	Hydrocele testis	NR
Jibiki et al. [8] 9	59	5	12	2 × IVIG (1 g/kg), ASA	Hands and feet	None	13 (right)	Hydrocele testis	Surgery 15 months later
10	19	6	4	4 × IVIG (1 g/kg), ASA, PSL	Hands and feet	13 (RCA, LCA, medium aneurysm)	5 (bilateral)	Hydrocele testis	Resolved 2 months later
Roy and Chakrabartty [9] 11	5	5	no description	IVIG, ASA	Feet	NR (LAD dilation)	7 (bilateral)	Orchitis	Resolved 72 h after IVIG
Tripathy et al. [10] 12	5	4	10	IVIG (2 g/kg), ASA	Hands and feet	None	10 (bilateral)	Hydrocele testis	Resolved 48 h after IVIG
Tan et al. 2021 [11] 13	18	2	9	IVIG (2 g/kg), ASA	None	14 (LAD, small aneurysm)	8 (right)	Hydrocele testis	Resolved 6 weeks later
Liu et al. [12] 14	3	3	8	IVIG (2 g/kg), ASA	Hands and feet	None	5 (bilateral)	Scrotal swelling	Resolved 10 days later
Weitzen et al. [13] 15	24	4	6	IVIG (2 g/kg), ASA	Right‐hand, penis	None	4 (laterality NR)	Scrotal edema	Resolved 2 weeks later
Present cases 2025 16	49	5	4	2 × IVIG (2 g/kg), ASA, PSL, clopidogrel, candesartan	Hands	8 (RCA, LMT, LAD, LCX; medium aneurysms)	7 (right)	Scrotal swelling	Resolved 1 month later

*Note:* IVIG, intravenous immunoglobulin; PSL, prednisolone; LAD, left anterior descending artery. This table was partially adapted from Jibiki et al. [8], with modifications by the authors.

Abbreviations: ASA, acetylsalicylic acid; LCA, left coronary artery; LCX, left circumflex artery; NR, not reported; RCA, right coronary artery.

The development of CAAs in children with KD is driven by acute vascular inflammation. During the acute phase, mononuclear cells, lymphocytes, and macrophages infiltrate the arterial walls. This infiltration leads to destruction of the internal elastic lamina, necrosis of smooth muscle cells, and proliferation of intimal myofibroblasts, ultimately resulting in coronary artery dilatation or aneurysm formation [2]. In the present case, although the patient initially responded to the first IVIG dose with defervescence, he subsequently developed acute scrotal involvement accompanied by progression of CAAs. This clinical course suggests that acute scrotal symptoms may reflect persistent disease activity. In our review, acute scrotal involvement was observed both before the diagnosis of KD and after treatment initiation, as in the present case (Table 1). Among the 16 previously reported KD cases with acute scrotal involvement, six developed CAAs despite treatment, although treatment regimens varied considerably across cases (Table 1). Therefore, acute scrotal symptoms may represent a potential clinical clue to persistent disease activity and should prompt careful reassessment for evolving coronary artery involvement.

Although further studies are needed, acute scrotal involvement may represent a potential clinical clue to persistent disease activity in KD. Recognition of this finding may prompt careful reassessment for evolving coronary artery involvement. Persistent or recurrent fever after initial treatment of KD warrants careful reassessment for persistent disease activity, while also considering concomitant bacterial infection and other inflammatory disorders in the differential diagnosis.

## Author Contributions

Dr. Yuma Inui collected clinical data, drafted the initial manuscript, and reviewed and revised the manuscript.

Dr. Katsuyuki Yokoi supervised case management and contributed to the drafting and critical revision of the manuscript.

Chihaya Sumida performed the initial outpatient evaluation of the patient and reviewed the manuscript.

Dr. Masato Itano contributed to the interpretation of clinical data, provided important intellectual input, and critically reviewed the manuscript.

Dr. Natsuki Hatakawa conducted a comprehensive literature review and contributed to the interpretation of the previously reported cases.

Dr. Takanori Suzuki provided expert clinical input and contributed to critical revision of the manuscript.

Dr. Hidetoshi Uchida managed the patient during hospitalization and contributed to the acquisition of clinical information.

Dr. Kazuyoshi Saito performed and interpreted cardiac imaging studies, including echocardiography and coronary CT, and critically reviewed the manuscript.

Dr. Yoshiki Kawamura provided senior supervision and validated final clinical interpretations.

## Funding

The authors did not receive support from any organization for the submitted work.

## Disclosure

All authors have read and approved the final version of the manuscript and agreed to be accountable for all aspects of the work.

## Ethics Statement

Ethical approval was not required for this case report, in accordance with institutional guidelines.

## Consent

Written permission was obtained from the patient’s guardian to publish this case and the corresponding photos or imaging studies.

## Conflicts of Interest

The authors declare no conflicts of interest.

## Data Availability

Data used to support the findings of this study are available on request from the corresponding author.
